# Clinically Significant Neuroimaging Findings Among Pediatric Patients Presenting to the Emergency Department With Symptoms of Psychosis: A Multicenter Retrospective Study

**DOI:** 10.1111/acem.70155

**Published:** 2025-09-30

**Authors:** Jennifer A. Hoffmann, Tapan K. Parikh, Doug Lorenz, Michael P. Goldman, Emily M. Powers, Shilpa J. Patel, Ilana S. Lavina, Theodore W. Heyming, Jasmin T. England, Mohsen Saidinejad, Ilene Claudius, Pallavi Ghosh, Daniel J. Shapiro, Tricia B. Swan, Kamali L. Bouvay, Eileen Murtagh Kurowski, Nadine M. Smith, Justin R. Davis, Alexander B. Moxam, Eli J. Muhrer, Rohit P. Shenoi, Elyse N. Portillo, Ron L. Kaplan, Neil G. Uspal, Robert M. Lapus, Andrea T. Vo, Daniel B. Fenster, Danielle B. Barrocas, Deborah R. Liu, Pradip P. Chaudhari, Rachel Cafferty, Stephen B. Freedman, Jerri A. Rose, Megan F. Evers, Ashley M. Metcalf, Fareed Saleh, Jennifer Dunnick, Raymond D. Pitetti, Yashas R. Nathani, Muhammad Waseem, Todd A. Florin, the Pediatric Emergency Medicine Collaborative Research Committee (PEMCRC) Psychosis Study Group

**Affiliations:** ^1^ Division of Emergency Medicine Ann & Robert H. Lurie Children's Hospital of Chicago Chicago Illinois USA; ^2^ Department of Pediatrics Northwestern University Feinberg School of Medicine Chicago Illinois USA; ^3^ Pritzker Department of Psychiatry and Behavioral Health Ann & Robert H. Lurie Children's Hospital of Chicago Chicago Illinois USA; ^4^ Department of Bioinformatics and Biostatistics, School of Public Health and Information Sciences University of Louisville Louisville Kentucky USA; ^5^ Yale University School of Medicine New Haven Connecticut USA; ^6^ Division of Emergency Medicine Children's National Hospital Washington DC USA; ^7^ Department of Emergency Medicine Children's Hospital of Orange County Orange California USA; ^8^ The Lundquist Institute for Biomedical Innovation at Harbor UCLA Medical Center David Geffen School of Medicine at UCLA Los Angeles California USA; ^9^ Harbor‐UCLA, David Geffen School of Medicine at UCLA Los Angeles California USA; ^10^ Children’s Healthcare of Atlanta Atlanta GA USA; ^11^ Division of Pediatric Emergency Medicine University of California San Francisco CA USA; ^12^ AdventHealth Ocala Ocala Florida USA; ^13^ TriHealth Group Health Pediatrics and Cincinnati Children’s Hospital Medical Center Cincinnati Ohio USA; ^14^ Cincinnati Children’s Hospital Medical Center Cincinnati Ohio USA; ^15^ Advocare Marlton Pediatrics Marlton NJ USA; ^16^ Department of Pediatrics University of Mississippi Medical Center Jackson Mississippi USA; ^17^ Department of Psychiatry University of Pennsylvania Philadelphia PA USA; ^18^ Charlie Health Bozeman MT USA; ^19^ Division of Emergency Medicine, Department of Pediatrics Baylor College of Medicine Houston Texas USA; ^20^ Division of Pediatric Emergency Medicine Baylor College of Medicine, Texas Children's Hospital Houston Texas USA; ^21^ Department of Pediatrics, Division of Emergency Medicine University of Washington School of Medicine, Seattle Children's Hospital Seattle Washington USA; ^22^ Seattle Children's Hospital University of Washington School of Medicine Seattle Washington USA; ^23^ McGovern Medical School at UTHealth Houston Houston Texas USA; ^24^ Texas Children's Hospital Houston TX USA; ^25^ Department of Emergency Medicine Columbia University Irving Medical Center New York New York USA; ^26^ Columbia University Irving Medical Center New York New York USA; ^27^ Children's Hospital Los Angeles Keck School of Medicine at University of Southern California Los Angeles California USA; ^28^ Department of Pediatrics, Section of Emergency Medicine University of Colorado School of Medicine Aurora Colorado USA; ^29^ Departments of Pediatrics and Emergency Medicine, Cumming School of Medicine University of Calgary Calgary Alberta Canada; ^30^ University Hospitals‐Rainbow Babies & Children's Hospital, Case Western Reserve School of Medicine Cleveland Ohio USA; ^31^ Rady Children's Hospital San Diego San Diego California USA; ^32^ Department of Pediatrics University of California‐San Diego San Diego California USA; ^33^ Department of Pediatrics, Division of Pediatric Emergency Medicine Duke University Hospital Durham NC USA; ^34^ Cook Children's Medical Center Forth Worth TX USA; ^35^ Lincoln Medical Center Bronx New York USA

## Abstract

**Background:**

The clinical utility of diagnostic neuroimaging for pediatric patients presenting to the emergency department (ED) for psychosis remains unclear. We sought to estimate the prevalence of and characteristics associated with clinically significant neuroimaging findings among pediatric patients presenting to the ED with symptoms of psychosis who had neuroimaging performed.

**Methods:**

This retrospective cross‐sectional study included visits by patients 5 to < 18 years old presenting with symptoms of psychosis to 28 EDs affiliated with the Pediatric Emergency Medicine Collaborative Research Committee from 2016 to 2019 and had neuroimaging performed. We estimated the rate of clinically significant neuroimaging findings, defined as those resulting in further testing, treatment, or medical admission, overall and by imaging modality. Multivariable logistic regression models examined presenting features associated with clinically significant findings.

**Results:**

Clinically significant neuroimaging findings were identified in 5.4% (95% CI 4.2%, 6.9%) of 1118 ED visits (54% male, median [IQR] 14 [11–16] years old). Clinically significant findings occurred in 4.9% (34/699) of head computed tomography scans and 7.5% (45/604) of brain magnetic resonance imaging studies (*p* = 0.07). In a model that imputed missing data, no presenting features were associated with clinically significant neuroimaging findings. In a model that treated missing documentation as absence of the clinical feature, the adjusted odds of clinically significant neuroimaging findings were lower among ED visits by patients with suspected alcohol or substance use (aOR 0.38, 95% CI 0.16, 0.87).

**Conclusion:**

Among pediatric patients presenting to the ED with symptoms of psychosis who had neuroimaging obtained, approximately 1 in 20 had clinically significant findings. Suspected alcohol or substance use was associated with lower odds of clinically significant neuroimaging findings, although this finding was not consistent across modeling approaches. Prospective studies are needed to definitively evaluate the utility of neuroimaging among children and adolescents presenting to the ED with symptoms of psychosis.

Schizophrenia and psychotic mood disorders affect 2% of the population, causing a high burden of disability, with peak onset in late adolescence and early adulthood [1, 2]. Children and adolescents with symptoms of psychosis are increasingly evaluated in emergency department (ED) settings. Pediatric ED visits for psychosis increased by 38% from 2012 to 2016 [3], and continued to increase beyond expected rates during the COVID‐19 pandemic [4]. When patients present to the ED for symptoms of psychosis, clinicians must first identify whether a non‐psychiatric condition accounts for their presentation [5, 6]. A variety of neurologic, infectious, rheumatologic, and other medical conditions can present with psychotic symptoms [7]. Neuroimaging, specifically brain magnetic resonance imaging (MRI) and head computed tomography (CT), is often obtained to evaluate for these conditions [8, 9]. The use of brain MRI for evaluation of children with psychosis has increased over time, yet there remains significant variation in its use across centers [9].

Prior studies have suggested a low diagnostic yield of neuroimaging for the evaluation of psychosis, although limitations in these prior works make definitive conclusions challenging. A systematic review of 16 studies that included 2312 patients with first‐episode psychosis found that structural abnormalities on neuroimaging rarely required clinical intervention (median: 3.5%) and were very rarely the cause of psychotic symptoms [10]. However, most patients in these studies were adults, and findings specific to pediatric patients were not reported separately. In a single‐center, retrospective study of approximately 400 patients aged 5–20 years who received a head CT in the ED for evaluation of acute psychosis or hallucinations, no studies had actionable findings [11]. However, this study did not examine the diagnostic yield of MRI, which may be more sensitive than head CT to detect subtle abnormalities, and is the preferred imaging modality recommended by the American Psychiatric Association for evaluation of psychosis [12]. Among 128 adolescents admitted to an inpatient psychiatric unit for psychosis, 7.0% had brain MRI findings that required further evaluation or management, while another small retrospective study completed in an inpatient psychiatric unit identified no actionable neuroimaging findings [13, 14]. The results from these studies may not generalize to the ED setting, where more patients may have non‐psychiatric conditions. In sum, prior work has not focused specifically on the population of children and adolescents presenting to the ED and has not investigated the breadth of neuroimaging modalities currently used in clinical practice. Moreover, prior studies have not evaluated which clinical features are associated with abnormal neuroimaging findings among pediatric patients presenting to the ED for symptoms of psychosis.

In a large multicenter cohort of children and adolescents presenting to the ED with symptoms of psychosis who had neuroimaging performed, we aimed to estimate the prevalence of clinically significant neuroimaging findings, defined as those resulting in further testing, treatment, or medical admission, and to identify clinical characteristics associated with clinically significant neuroimaging findings.

## 1 Methods

### 1.1 Study Design, Setting, and Population

This was a multicenter retrospective cross‐sectional study of visits to 28 EDs in the US and Canada by patients 5 to < 18 years old who presented with symptoms of psychosis and had neuroimaging performed between January 1, 2016 and December 31, 2019. Participating EDs were in 14 US states, the District of Columbia, and 2 Canadian provinces. Twenty‐two EDs were in children's hospitals and 6 in non‐children's hospitals. Data were collected from site electronic health records (EHRs). The study was endorsed by the Pediatric Emergency Medicine Collaborative Research Committee of the American Academy of Pediatrics Section on Emergency Medicine and was approved by the institutional review boards at participating sites. The study followed the Strengthening the Reporting of Observation Studies in Epidemiology (STROBE) reporting guideline [15].

Eligible ED visits were those in which patients 5 to < 18 years old presented for symptoms of psychosis, defined as delusions, hallucinations, disorganized speech, disorganized behavior, or catatonia [16], and had neuroimaging performed during or within 7 calendar days of the visit. Neuroimaging was defined as a head CT, complete brain MRI, or an abbreviated MRI brain protocol such as a “rapid brain” or “ventricle” study. If a neuroimaging study was obtained at another ED prior to transfer to the study site, the visit was included if a complete neuroimaging report was available. If a patient had multiple ED visits with neuroimaging performed within 7 days, only the first ED visit was included. Exclusions were: (1) pre‐existing central nervous system disease, defined by a known medical condition that results in abnormal neuroimaging; or (2) non‐verbal when in the usual state of health.

Cases were identified via a three‐step process. First, ED visits by patients < 18 years old were identified at each site that had: (1) a chief complaint categorized as mental health‐related (e.g., “psychiatric” or “behavioral health”); (2) a keyword in the ED clinician note of “psychosis,” “psychotic,” “hallucination,” “voices,” “delusion,” “bizarre,” “catatonia,” “catatonic,” or spelling variants; or (3) any ICD‐10‐CM diagnosis code for schizophrenia spectrum or psychotic disorders, bipolar and related disorders, dissociative disorders, hallucinations, poor hygiene, or bizarre appearance or behavior (Table S1) [17, 18]. Some sites did not have the capability to search chief complaint categories (if chief complaints were entered as free text only) or to perform keyword searches. Of the 28 participating EDs, 13 used all three identification strategies, 8 EDs used chief complaint category and diagnosis codes, and 7 EDs used diagnosis codes only. Second, visits by patients ≥ 5 years old that had neuroimaging performed during or within 7 calendar days of the visit were retained. Third, for the remaining visits, site investigators trained in pediatric emergency medicine performed manual review of ED clinician notes to assess eligibility based on inclusion and exclusion criteria.

### 1.2 Data Collection

Following best practices for EHR reviews [19, 20], site investigators were trained and provided with a detailed manual of operations with strict definitions for data collection. The trained investigators abstracted data into a centralized, standardized REDCap data collection form [21, 22]. Physician site investigators entered data elements related to symptoms of psychosis, clinically significant findings, and whether findings were likely related to symptoms of psychosis. At some sites, specific data elements (e.g., demographics, laboratory results) were entered by trained non‐physician research assistants or nurses and verified by site investigators trained in pediatric emergency medicine.

EHRs were reviewed to ascertain demographic, historical, and physical examination findings; laboratory and neuroimaging findings; and disposition. When abstracting clinician‐documented data, a hierarchical approach was taken; in cases of conflicting data, data recorded by the most senior clinician were used [23]. If historical or physical examination data were missing in ED notes, the admission note was used as a secondary source of information, when available. Laboratory data were obtained from the ED visit and, if an admission occurred at the same facility, during the associated hospitalization.

Of included ED visits, 5%–10% were reviewed by a second trained investigator at each site for the purpose of assessing interrater reliability of data abstraction from medical records.

### 1.3 Study Measures

The primary outcome was the presence of clinically significant neuroimaging findings, defined by whether the findings resulted in further testing, treatment, or medical admission, using a previously developed standardized scheme (Table S2) [24]. If multiple neuroimaging studies were obtained, the visit was considered to have clinically significant neuroimaging findings if relevant findings appeared on any individual study. A secondary outcome was the presence of neuroimaging findings that were “likely related to psychosis,” defined as findings that were meaningful in elucidating the etiology of psychosis, managing symptoms, or determining treatment strategies.

Demographic variables included age, sex, race, and ethnicity as designed in the EHR. Presenting symptoms of psychosis were categorized as hallucinations, delusions, disorganized speech, disorganized behavior, and catatonia. First‐episode psychosis was defined as either having no documented prior healthcare visits for symptoms of psychosis, or only having documented visits within 1 month of the index ED visit, which were considered part of the same episode. The following clinical features were considered “present” if they occurred during or within 1 week of the ED visit: amnesia, fever reported by history, head trauma, headache, seizure‐like activity, tired/lethargic, vomiting, weakness, sensory changes, vision changes, and gait changes. Alcohol/substance use was considered “present” if suspected within 24 h of the ED visit. History of neurologic and/or psychiatric conditions was recorded when documented. Physical examination findings included: fever measured during the ED visit, tachycardia (defined by Pediatric Advanced Life Support criteria [25]) upon first recorded vital signs, altered consciousness, meningismus, and focal neurologic deficit. For each laboratory study, the first interpretable result was recorded. For each neuroimaging study, data included the location obtained (ED, medical unit, psychiatric unit) and finding category (normal, normal variant, infectious, inflammatory, structural, other). Disposition was categorized as admission to a medical unit, admission/transfer to a psychiatric unit, discharge home, and other.

### 1.4 Analysis

Demographic and clinical characteristics were summarized using descriptive statistics. For each variable, concordance between data abstractors was compared using Cohen's kappa. With 95% confidence intervals, we estimated rates of: (1) visits by patients with clinically significant neuroimaging findings, (2) visits by patients with neuroimaging findings likely related to psychosis, and (3) visits by patients with neuroimaging findings that were both clinically significant and likely related to psychosis. Based on pilot data from one site, we anticipated identifying 1800 eligible ED visits and 144 visits with clinically significant neuroimaging findings, enabling estimation of the prevalence of clinically significant neuroimaging findings with a 95% CI width of ±1.3%.

Among ED visits for first‐episode psychosis, a planned subgroup analysis was performed to estimate rates of neuroimaging findings that were clinically significant and/or likely related to psychosis.

A post hoc subgroup analysis was performed to estimate rates of neuroimaging findings that were clinically significant and/or likely related to psychosis among patients without presenting signs or symptoms that might independently prompt neuroimaging. This list of signs and symptoms was based on criteria indicating “a possible need for neuroimaging” in the American Psychiatric Association Practice Guideline for the Treatment of Patients with Schizophrenia [12], prior literature examining neuroimaging findings in children with psychosis [11], and the clinical experience of the authorship group, which included practicing pediatric emergency medicine physicians. These signs and symptoms were: head trauma, headache, seizure‐like activity, amnesia, vomiting, tired/lethargic, weakness, sensory change, vision change, gait change, altered consciousness, meningismus, and focal neurologic deficit.

Using linear contrasts of model coefficients derived from logistic regression, we compared (1) rates of clinically significant neuroimaging findings found via head CT and brain MRI and (2) rates of clinically significant neuroimaging found on complete brain MRI protocols and abbreviated MRI protocols. We estimated, with 95% confidence intervals, the rates of clinically significant abnormal neuroimaging by the location where neuroimaging was obtained.

We developed logistic regression models to identify clinical features associated with clinically significant neuroimaging findings. Clinical features were selected from demographic, historical, and physical exam findings. Laboratory test results were not considered in models due to high rates of missingness (each laboratory test was not obtained in > 10% of visits). Multivariable model building was accomplished through the following process. We did not incorporate race and ethnicity into models as they do not reflect biological differences, but rather social constructs [26]. Next, we grouped clinical features with very low prevalence (positive in < 5% of the study sample) into larger categories that had shared clinical relevance. Specifically, we grouped fever by history or in the ED, and we grouped all neurologic signs and symptoms. Reference groups were chosen to reflect the largest categories in the study sample. Including random intercepts per hospital did not appreciably improve model fit, so these were not incorporated in the final models. Two models were developed, each with different approaches for handling missing data. In the first model, missing data were imputed by the multiple imputation by chained equations algorithm, producing 5 imputed data sets over which models were fit and results imputation‐averaged [27]. In the second model, clinical features that were not documented in the health record were analyzed as absent, as we presumed that missing documentation likely disproportionately reflected the absence of the clinical feature [20, 28]. Models were fit by penalized maximum likelihood with optimal penalty selected by systematic search to mitigate the relatively high number of predictors and low number of cases with clinically significant neuroimaging findings. Results were reported as unadjusted and adjusted odds ratios (aORs) with 95% confidence intervals.

A planned subgroup analysis was performed to examine characteristics associated with clinically significant neuroimaging findings during visits for first‐episode psychosis. The same approach as above was used to construct these regression models.

Analyses were performed using R Statistical Software version 4.3.1 (R Foundation), with add‐on libraries *mice* and *rms* [29, 30].

## 2 Results

### 2.1 Characteristics of Study Sample

**FIGURE 1:**
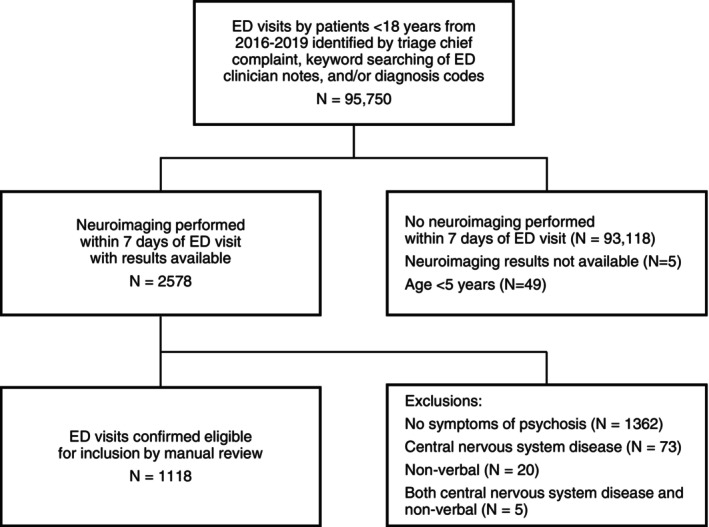
Flow diagram for case inclusion/exclusion. ED, emergency department.

We included 1118 ED visits by pediatric patients with symptoms of psychosis who had neuroimaging performed (Figure 1). During these visits, 1325 unique neuroimaging studies were performed. Of included visits, 54% were by males, the median age was 14 years (interquartile range 11–16 years), 41% resulted in admission to a medical unit, and 33% resulted in admission or transfer to a psychiatric unit (Table 1). There were 631 ED visits for first‐episode psychosis.

**TABLE 1:** Demographic and clinical characteristics of emergency department visits by children and adolescents presenting with symptoms of psychosis with neuroimaging performed.

Characteristic	Visits *N*, %	Missing; unknown; *N* (%)	No significant neuroimaging findings^a^; *N* (%)	Significant neuroimaging findings^a^; *N* (%)	Percent difference (95% CI)
Overall	*N* = 1118		*N* = 1058	*N* = 60	
Age					
5–10	221 (20%)	0 (0%)	200 (19%)	21 (35%)	16 (4, 28)
11–14	351 (31%)		333 (31%)	18 (30%)	‐1 (−13, 10)
15–17	546 (49%)		525 (50%)	21 (35%)	−15 (−27, −2)
Male sex	609 (54%)	0 (0%)	572 (54%)	37 (62%)	8 (−5, 20)
Race/Ethnicity					
Hispanic	273 (27%)	92 (8%)	263 (27%)	10 (19%)	−8 (−19, 3)
Non‐Hispanic Asian	56 (5%)		52 (5%)	4 (8%)	2 (−5, 9)
Non‐Hispanic Black	315 (31%)		298 (31%)	17 (32%)	1 (−11, 14)
Non‐Hispanic White	335 (33%)		315 (32%)	20 (38%)	5 (−8, 19)
Other	47 (5%)		45 (5%)	2 (4%)	−1 (−6, 4)
Symptoms of psychosis					
Hallucinations	786 (77%)	100 (9%)	751 (78%)	35 (67%)	−10 (−23, 2)
Delusions	298 (34%)	241 (22%)	284 (34%)	14 (33%)	−1 (−14, 11)
Disorganized speech	303 (31%)	125 (11%)	285 (30%)	18 (35%)	4 (−8, 17)
Disorganized behavior	575 (56%)	82 (7%)	541 (55%)	34 (60%)	4 (−8, 17)
Catatonia	87 (9%)	129 (12%)	80 (9%)	7 (13%)	5 (−4, 13)
Clinical history					
Head trauma	55 (7%)	286 (26%)	53 (7%)	2 (5%)	−1 (−7, 4)
Headache	311 (32%)	152 (14%)	292 (32%)	19 (37%)	5 (−7, 18)
Seizure‐like activity	86 (10%)	214 (19%)	78 (9%)	8 (18%)	9 (−1, 19)
Amnesia	60 (9%)	421 (38%)	58 (9%)	2 (8%)	−1 (−8, 6)
Vomiting	84 (8%)	115 (10%)	77 (8%)	7 (13%)	5 (−4, 14)
Tired/Lethargic	137 (15%)	220 (20%)	124 (14%)	13 (31%)	16 (5, 28)
Fever (by history)	47 (4%)	66 (6%)	40 (4%)	7 (12%)	8 (0, 17)
Alcohol/Substance use	173 (19%)	221 (20%)	167 (20%)	6 (14%)	−5 (−14, 4)
First‐episode psychosis	631 (71%)	224 (20%)	586 (70%)	45 (83%)	14 (4, 23)
Weakness	24 (3%)	223 (20%)	23 (3%)	1 (2%)	0 (−4, 3)
Sensory	34 (4%)	329 (29%)	31 (4%)	3 (9%)	5 (−2, 12)
Vision change	78 (10%)	302 (27%)	71 (9%)	7 (21%)	12 (1, 22)
Gait change	66 (8%)	254 (23%)	57 (7%)	9 (20%)	14 (3, 24)
Past history					
Neurologic condition	116 (11%)	29 (3%)	111 (11%)	5 (8%)	−2 (−10, 5)
Psychiatric condition	534 (49%)	34 (3%)	514 (50%)	20 (33%)	−17 (−29, −5)
Physical exam					
Fever during visit	27 (2%)	6 (1%)	24 (2%)	3 (5%)	3 (−3, 8)
Tachycardia	265 (24%)	10 (1%)	248 (24%)	17 (29%)	6 (−6, 17)
Altered consciousness	193 (17%)	6 (1%)	179 (17%)	14 (23%)	6 (−5, 17)
Meningismus	4 (0%)	38 (3%)	4 (0%)	0 (0%)	0 (−1, 0)
Focal neurologic deficit	31 (3%)	23 (2%)	25 (2%)	6 (10%)	8 (0, 15)
Disposition					
Admitted to medical unit	461 (41%)	0 (0%)	421 (40%)	40 (67%)	27 (15, 39)
Admitted/Transferred to psychiatric unit	369 (33%)		358 (34%)	11 (18%)	−16 (−26, −5)
Discharged home	277 (25%)		269 (25%)	8 (13%)	−12 (−21, −3)
Other	11 (1%)		10 (1%)	1 (2%)	1 (−3, 4)

^a^ Percentages exclude missing values.

### 2.2 Interrater Reliability of Neuroimaging Categorization

The Kappa statistic for categorization of clinical significance was 0.79 (0.51, 1.0), indicating substantial agreement across data abstractors. The Kappa statistic for categorization of findings likely related to psychosis was 0.86 (0.58, 1.0), indicating substantial agreement across data abstractors.

### 2.3 Rates of Visits With Clinically Significant Neuroimaging Findings

Of 1118 ED visits with neuroimaging performed, 60 visits (5.4%; 95% CI 4.2%, 6.9%) were by patients with clinically significant neuroimaging findings, 38 visits (3.4%; 95% CI 2.4%, 4.7%) were by patients with neuroimaging findings likely related to psychosis, and 27 visits (2.4%; 95% CI 1.6%, 3.5%) were by patients with neuroimaging findings that were both clinically significant and likely related to psychosis.

Of 631 ED visits for first‐episode psychosis, 45 visits (7.1%; 95% CI 5.3%, 9.5%) were by patients with clinically significant neuroimaging findings, 29 visits (4.6%; 95% CI 3.2%, 6.6%) were by patients with neuroimaging findings likely related to psychosis, and 22 visits (3.4%; 95% CI 2.3%, 5.3%) were by patients with neuroimaging findings that were both clinically significant and likely related to psychosis.

In the subgroup of 476 ED visits without any specified signs or symptoms that might independently prompt neuroimaging, 20 visits (4.2%; 95% CI 2.7%, 6.5%) were by patients with clinically significant neuroimaging findings, 9 visits (1.9%; 95% CI 0.9%, 3.7%) were by patients with neuroimaging findings likely related to psychosis, and 4 visits (0.8%; 95% CI 0.3%, 2.3%) were by patients with neuroimaging findings that were both clinically significant and likely related to psychosis.

### 2.4 Clinically Significant Findings by Imaging Modality and Location

Of 1325 unique neuroimaging studies performed, 80 had clinically significant findings: 16 (20%) were infectious, 18 (23%) were inflammatory, 45 (56%) were structural, and 9 (11%) had other findings (Tables S3 and S4). There were 699 non‐contrast head CTs, 604 brain MRIs (complete and abbreviated), and 22 other studies performed (Table 2).

**TABLE 2:** Neuroimaging studies among children presenting to the emergency department with symptoms of psychosis.

Characteristic	All studies^a^	Non‐contrast; head CT; *N* (%)	Complete; brain MRI; *N* (%)	Abbreviated brain MRI; *N* (%)	Other CT; *N* (%)	Other MRI *N* (%)
Overall	*N* = 1325	*N* = 699	*N* = 577	*N* = 27	*N* = 2	*N* = 20
Location obtained						
Emergency department	807 (61%)	649 (93%)	140 (24%)	17 (63%)	0 (0%)	1 (5%)
Medical unit	278 (21%)	28 (4%)	243 (42%)	2 (7%)	1 (50%)	4 (20%)
Psychiatric unit	209 (16%)	17 (2%)	190 (33%)	2 (7%)	0 (0%)	0 (0%)
Imaging findings						
Normal	1012 (76%)	582 (83%)	394 (68%)	23 (85%)	2 (100%)	11 (55%)
Normal variant	149 (11%)	46 (7%)	97 (17%)	3 (11%)	0 (0%)	3 (15%)
Infectious	41 (3%)	21 (3%)	17 (3%)	1 (4%)	0 (0%)	2 (10%)
Inflammatory	37 (3%)	12 (2%)	22 (4%)	0 (0%)	0 (0%)	3 (15%)
Structural	100 (8%)	42 (6%)	55 (10%)	0 (0%)	0 (0%)	3 (15%)
Other	33 (2%)	11 (2%)	21 (4%)	0 (0%)	0 (0%)	1 (5%)
Clinically significant finding	80 (6%)	34 (5%)	44 (8%)	1 (4%)	0 (0%)	1 (5%)
Relation to psychosis						
Likely related	57 (4%)	20 (3%)	33 (6%)	1 (4%)	0 (0%)	3 (15%)
Normal/Likely unrelated	1227 (93%)	654 (94%)	528 (92%)	26 (96%)	2 (100%)	17 (85%)
Unclear	41 (3%)	25 (4%)	16 (3%)	0 (0%)	0 (0%)	0 (0%)

Abbreviations: CT, computed tomography; MRI, magnetic resonance imaging.

^a^ Counts represent individual neuroimaging studies rather than ED visits. Some ED visits had more than one neuroimaging study performed.

Clinically significant findings occurred in 4.9% (95% CI 3.4%, 6.8%) of non‐contrast head CTs and in 7.5% of brain MRIs (95% CI 5.5%, 9.9%, *p* = 0.07). Clinically significant findings occurred in 7.7% (95% CI 5.7%, 10.2%) of complete brain MRI protocols and 3.7% of abbreviated brain MRI protocols (95% CI 0.2%, 21.0%, *p* = 0.75).

Rates of clinically significant abnormal neuroimaging were 5.3% (95% CI 3.9%, 7.2%) in the ED, 5.0% (95% CI 2.9%, 8.5%) after admission to a medical unit, and 10.5% (95% CI 6.9%, 15.7%) when obtained after admission to a psychiatric unit (*p* = 0.01).

### 2.5 Clinical Features Associated With Clinically Significant Neuroimaging Findings

Kappa statistics for abstraction of clinical features are shown in Table S5. In a univariate model using imputation for missing data, the odds of clinically significant neuroimaging findings were higher for visits by patients 5–10 years old (compared with 15–17 years old); higher for visits by patients with lethargy, fever, neurologic signs/symptoms, and first‐episode psychosis; and lower for visits by patients with a history of a psychiatric condition. In a univariate model that treated missing documentation as absence of the clinical feature, the odds of clinically significant neuroimaging findings were higher for visits by patients 5–10 years old (compared with 15–17 years old); higher for patients with lethargy, fever, neurologic signs/symptoms, and first‐episode psychosis; and lower for visits by patients with hallucinations and a history of a psychiatric condition (Table S6).

**FIGURE 2:**
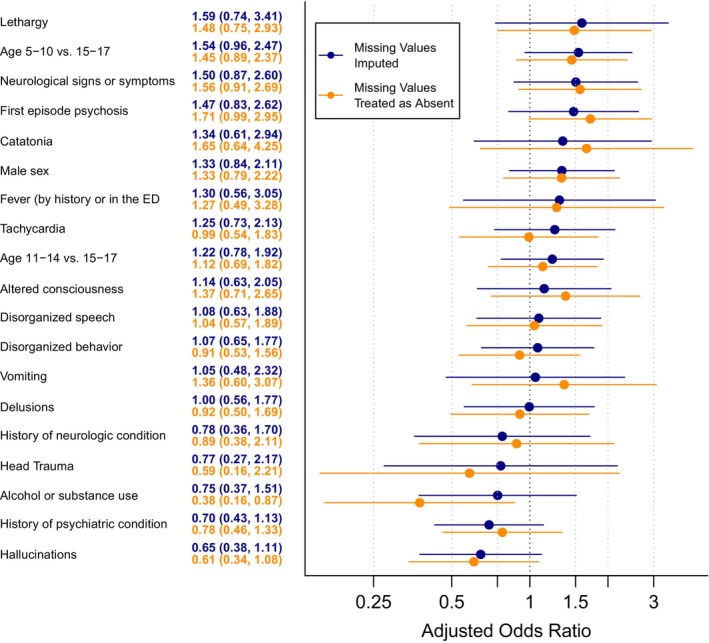
Factors associated with clinically significant neuroimaging among children and adolescents presenting to the emergency department with psychosis. In the first model, imputation was used to handle missing data. In the second model, missing values (with no documentation of the clinical finding within clinician notes) were treated as absent. Neurological signs or symptoms included: Headache, seizure, amnesia, weakness, sensory, vision change, gait change, meningismus, and/or focal neurologic deficit.

In a multivariable model using imputation for missing data, no clinical features were associated with clinically significant neuroimaging findings. In a multivariable model that treated missing documentation as absence of the clinical feature, the adjusted odds of clinically significant neuroimaging were lower among ED visits with suspected alcohol or substance use (aOR 0.38, 95% CI 0.16, 0.87) (Figure 2).

**FIGURE 3:**
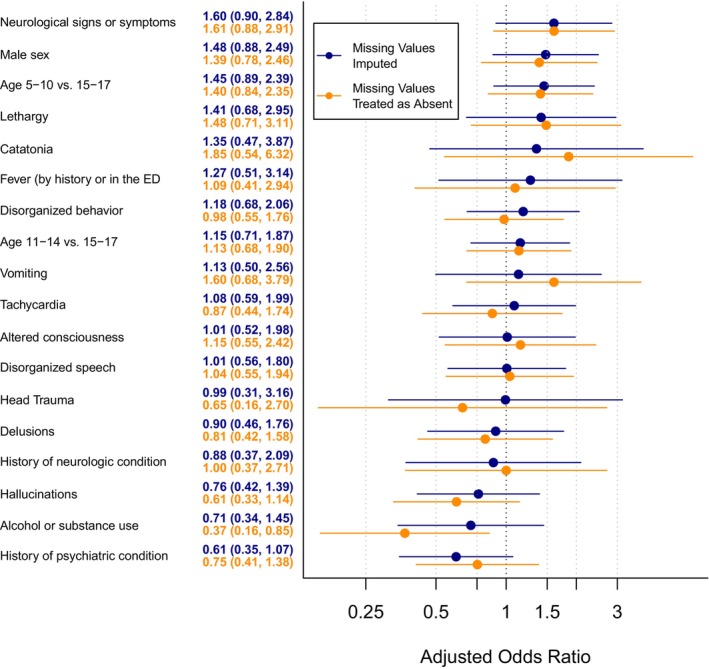
Factors associated with clinically significant neuroimaging among children and adolescents presenting to the emergency department with first‐episode psychosis. In the first model, imputation was used to handle missing data. In the second model, missing values (with no documentation of the clinical finding within clinician notes) were treated as absent. Neurological signs or symptoms included: Headache, seizure, amnesia, weakness, sensory, vision change, gait change, meningismus, and/or focal neurologic deficit. ED: Emergency department.

In the subgroup of ED visits for first‐episode psychosis, in a multivariable model using imputation for missing data, no clinical features were associated with clinically significant neuroimaging findings. In a multivariable model that treated missing documentation as absence of the clinical feature, the adjusted odds of clinically significant neuroimaging were lower among ED visits with suspected alcohol or substance use (aOR 0.37, 95% CI 0.16, 0.85) (Figure 3).

## 3 Discussion

In this multicenter retrospective analysis of ED visits by children and adolescents who presented with symptoms of psychosis and had neuroimaging obtained, approximately 1 in 20 visits had clinically significant neuroimaging findings. To our knowledge, our study includes the largest pediatric cohort of ED visits for symptoms of psychosis, allowing for more precise estimates of rare outcomes. In a model using imputation to handle missing data, no presenting clinical features were associated with clinically significant neuroimaging findings. In a model in which missing clinical data were treated as absence of that clinical finding, suspected alcohol or substance use was associated with lower odds of clinically significant neuroimaging findings. These findings were consistent among the subgroup of ED visits by patients presenting with first‐episode psychosis.

It is important for emergency medicine clinicians to diagnose treatable non‐psychiatric conditions among children and adolescents who present with symptoms of psychosis. A wide variety of medical disorders have been associated with symptoms of psychosis, such as genetic syndromes, autoimmune diseases, and brain malformations [31], some of which have characteristic neuroimaging findings. Thus, neuroimaging can contribute to a comprehensive diagnostic evaluation for symptoms of psychosis. Additionally, negative neuroimaging results can provide reassurance to families, which may facilitate acceptance of new psychiatric diagnoses in some cases [32]. Nevertheless, the potential benefits of neuroimaging must be balanced against risks. Drawbacks to obtaining neuroimaging include cost, prolonged length of stay, radiation exposure, sedation risks, iatrogenic risks posed by the discovery of incidental findings, and delays in emergent imaging for other patients in the ED [11, 33, 34].

In our large multicenter study, we found non‐negligible rates of clinically significant neuroimaging findings, which differ from several single‐center studies of pediatric patients presenting to the ED or admitted to the hospital for psychosis that identified no clinically significant neuroimaging findings [11, 35, 36, 37]. For instance, one single‐center study examined 397 head CTs obtained in a pediatric ED for an indication of hallucination, psychosis, psychotic, or hearing voices. After excluding patients with headache, vomiting, trauma, seizure, or focal neurologic deficit, no studies had actionable findings [11]. In another single‐center study of 111 adolescents (13–19 years old) consecutively admitted for first‐onset psychosis who had an unremarkable medical history and normal physical examination, no neuroimaging tests led to a previously unknown or unsuspected medical diagnosis or played an important role in the clinical care of the patient [37]. Among 115 patients age 12–30 years admitted to a psychiatric inpatient unit for psychosis, 101 head CTs and 22 brain MRIs were performed, with none revealing actionable findings [13]. Several factors may have contributed to the higher rate of clinically significant findings identified in our study relative to prior work. Our study included younger patients, did not exclude patients based on specific presenting symptoms such as vomiting or headache, and used a broader definition of clinical significance that encompassed a need for further testing, in addition to treatment and medical admission. When we examined a subgroup of ED visits in which patients had no signs or symptoms that might independently prompt neuroimaging, fewer than 1% of visits had neuroimaging findings that were both clinically significant and likely related to psychosis.

To our knowledge, our study is the first to examine presenting features associated with clinically significant neuroimaging findings among pediatric patients presenting to the ED. In the inpatient psychiatric setting, among 128 patients aged 10–17 years with psychosis in whom a brain MRI scan was acquired, 7.0% triggered clinical referrals for further evaluation or management, compared with a 1.6% clinical referral rate for brain MRIs obtained among healthy age‐ and gender‐matched controls [14]. Among the patients in the study with psychosis, clinical referral was not significantly associated with sex, diagnosis, history of perinatal complications, neurodevelopmental delays or deviations, psychiatric comorbidity, or drug use [14]. These data are largely consistent with our finding that few presenting clinical features are associated with having relevant neuroimaging findings.

In our study, the only presenting feature associated with lower odds of clinically significant neuroimaging findings was suspected alcohol or substance use. Among adolescents, substance‐induced psychosis is most strongly associated with cannabis use [38] but has also been described secondary to stimulants, hallucinogens, synthetic agents, alcohol, and sedatives/hypnotics [8]. At present, neuroimaging guidelines for patients presenting with psychosis do not incorporate suspected alcohol or substance use into decision algorithms [12, 39]. The American Psychiatric Association Practice Guideline for the Treatment of Patients with Schizophrenia states that the following factors suggest a possible need for neuroimaging: focal neurological signs, new onset of seizures, later age at symptom onset, symptoms suggestive of intracranial pathology (e.g., chronic or severe headaches, nausea, vomiting), and symptoms suggestive of autoimmune encephalitis (e.g., rapid progression of working memory deficits over < 3 months; decreased or altered level of consciousness, lethargy, or personality change) [12]. In our pediatric sample, we found that younger age, fever, lethargy, abnormal neurologic signs and symptoms, and first‐episode psychosis were associated with clinically significant neuroimaging findings in univariate models, but not in multivariable models. Because we did not identify any clinical features that were consistently associated with clinically significant neuroimaging findings across modeling approaches, we were unable to develop a clinical prediction model to inform neuroimaging choices.

This work provides foundational data for a future prospective study to evaluate the role of diagnostic testing for children and adolescents presenting to the ED with symptoms of psychosis. Additional studies are needed to determine if a universal imaging strategy is indicated for these patients, or if imaging can be targeted to patients presenting with certain presenting clinical features. Future directions should also consider the cost‐effectiveness of neuroimaging for this population [40]. Finally, beyond simply revealing alternative diagnoses, structural and functional neuroimaging biomarkers deserve further study to understand whether they can predict the clinical course and guide the selection of interventions for children and adolescents with psychosis [41, 42, 43].

### 3.1 Limitations

This study has limitations inherent to its retrospective design. The analysis was limited to encounters in which neuroimaging was obtained. Clinicians may be more likely to order neuroimaging when they anticipate positive findings, which may bias the results in favor of overestimating the prevalence of clinically significant findings. Conversely, the identification of ED visits using diagnosis codes for psychosis may have biased the results in favor of underestimating clinically significant findings. When neuroimaging uncovers a non‐psychiatric diagnosis, related diagnosis codes may be applied to visits, rather than diagnosis codes for psychosis. We attempted to reduce this bias by identifying cases based on chief complaints and keyword searches of notes, in addition to diagnosis codes, but not all sites had the capability to identify cases using these modalities. Additionally, we were limited by the accuracy and availability of clinical data recorded in the EHR. Radiology findings were based on EHR reports rather than independent review of original images. Data abstractors were not blinded to the results of the neuroimaging studies. Follow‐up was not uniform, as records were unavailable for patients transferred to other psychiatric facilities.

## 4 Conclusions

In this multicenter retrospective study of children and adolescents presenting to the ED with symptoms of psychosis who had neuroimaging obtained, approximately 1 in 20 ED visits had clinically significant neuroimaging findings, prompting further testing, treatment, or medical admission. Among visits in which patients had no signs or symptoms that might independently prompt neuroimaging, only approximately 1 in 50 visits had clinically significant neuroimaging findings. Univariate models found that younger age, abnormal neurologic signs and symptoms, and lethargy were associated with clinically significant neuroimaging findings, while some multivariable models identified that suspected alcohol or substance use was associated with lower odds of clinically significant neuroimaging findings. Inherent to the retrospective nature of this study, identified rates of clinically significant findings may have been over‐ or underestimated, precluding definitive conclusions. Prospective studies are needed to estimate rates of clinically significant neuroimaging findings and associated clinical features in order to inform clinical decisions about when to obtain neuroimaging for children and adolescents presenting to the ED with symptoms of psychosis.

## Author Contributions

Jennifer A. Hoffmann conceived and designed the work; contributed substantially to the acquisition, analysis, and interpretation of data; and drafted the manuscript. Doug Lorenz provided substantial contributions to study design, substantial contributions to analysis and interpretation of data, and revised the work critically for important intellectual content. Tapan K. Parikh, Michael P. Goldman, Emily M. Powers, Shilpa J. Patel, Ilana S. Lavina, Theodore W. Heyming, Jasmin T. England, Mohsen Saidinejad, Ilene Claudius, Pallavi Ghosh, Daniel J. Shapiro, Tricia B. Swan, Kamali L. Bouvay, Eileen Murtagh Kurowski, Nadine M. Smith, Justin R. Davis, Alexander B. Moxam, Eli J. Muhrer, Rohit P. Shenoi, Elyse N. Portillo, Ron L. Kaplan, Neil G. Uspal, Robert M. Lapus, Andrea T. Vo, Daniel B. Fenster, Danielle B. Barrocas, Deborah R. Liu, Pradip P. Chaudhari, Rachel Cafferty, Stephen B. Freedman, Jerri A. Rose, Megan F. Evers, Ashley M. Metcalf, Fareed Saleh, Jennifer Dunnick, Raymond D. Pitetti, Yashas R. Nathani, and Muhammad Waseem provided substantial contributions to the acquisition and interpretation of data, and revised the work critically for important intellectual content. Todd A. Florin provided substantial contributions to study conception and design, substantial contributions to interpretation of data, and revised the work critically for important intellectual content. All authors provided final approval of the version to be published and agree to be accountable for all aspects of the work in ensuring that questions related to the accuracy or integrity of any part of the work are appropriately investigated and resolved.

## Conflicts of Interest

Tapan K. Parikh reports direct/indirect research funding for industry sponsored clinical trials from Alkermes, AbbVie, MindMed, Compass Pathways, Karuna Therapeutics, Cerevel, LB pharmaceuticals, Lyndra Therapeutics, Merck, Neurocrine Biosciences, Intra‐Cellular Therapies, and Teva.

## Supporting information


**Data S1:** acem70155‐sup‐0001‐DataS1.docx.

## Data Availability

The authors have nothing to report.
