# Supplementary material for: Clinically Significant Neuroimaging Findings Among Pediatric Patients Presenting to the Emergency Department With Symptoms of Psychosis: A Multicenter Retrospective Study
Source: Acad Emerg Med. 2025 Sep 30;33(1):e70155. doi: 10.1111/acem.70155 (PMC12517762; doi:10.1111/acem.70155)
Supplement: Supplementary file 1 — Data S1: acem70155‐sup‐0001‐DataS1.docx. [file ACEM-33-0-s001.docx]

**Supplementary Material**

**Supplemental Table 1.** Diagnosis Codes used for Case Ascertainment

**Supplemental Table 2.** Scheme for Categorization of Clinical Significance

**Supplemental Table 3.** Brief Descriptions of Clinically Significant Neuroimaging Findings among Children and Adolescents Presenting to the Emergency Department with Symptoms of Psychosis

**Supplemental Table 4.** Detailed Descriptions of Clinically Significant Neuroimaging Findings among Children and Adolescents Presenting to the Emergency Department with Symptoms of Psychosis

**Supplemental Table 5.** Interrater Reliability of Data Abstraction of Clinical Characteristics

**Supplemental Table 6.** Factors Associated with Clinically Significant Neuroimaging among Children and Adolescents Presenting to the Emergency Department with Symptoms of Psychosis, Univariate Models

**Supplemental Table 1. Diagnosis Codes used for Case Ascertainment**

| **Mental Health Disorder Group^1^** | **Diagnosis Code** | **Description** |
| --- | --- | --- |
| Schizophrenia Spectrum and Other Psychotic Disorders | F06.0 | Psychotic disorder with hallucinations due to known physiological condition |
|  | F06.1 | Catatonic disorder due to known physiological condition |
|  | F20.0 | Paranoid schizophrenia |
|  | F20.1 | Disorganized schizophrenia |
|  | F20.2 | Catatonic schizophrenia |
|  | F20.3 | Undifferentiated schizophrenia |
|  | F20.5 | Residual schizophrenia |
|  | F20.81 | Schizophreniform disorder |
|  | F20.89 | Other schizophrenia |
|  | F20.9 | Schizophrenia, unspecified |
|  | F22 | Delusional disorders |
|  | F23 | Brief psychotic disorder |
|  | F24 | Shared psychotic disorder |
|  | F25.0 | Schizoaffective disorder, bipolar type |
|  | F25.1 | Schizoaffective disorder, depressive type |
|  | F25.8 | Other schizoaffective disorders |
|  | F25.9 | Schizoaffective disorder, unspecified |
|  | F29 | Unspecified psychosis not due to a substance or known physiological condition |
| Bipolar and Related Disorders | F30.10 | Manic episode without psychotic symptoms, unspecified |
|  | F30.11 | Manic episode without psychotic symptoms, mild |
|  | F30.12 | Manic episode without psychotic symptoms, moderate |
|  | F30.13 | Manic episode, severe, without psychotic symptoms |
|  | F30.2 | Manic episode, severe with psychotic symptoms |
|  | F30.3 | Manic episode in partial remission |
|  | F30.4 | Manic episode in full remission |
|  | F30.8 | Other manic episodes |
|  | F30.9 | Manic episode, unspecified |
|  | F31.0 | Bipolar disorder, current episode hypomanic |
|  | F31.10 | Bipolar disorder, current episode manic without psychotic features, unspecified |
|  | F31.11 | Bipolar disorder, current episode manic without psychotic features, mild |
|  | F31.12 | Bipolar disorder, current episode manic without psychotic features, moderate |
|  | F31.13 | Bipolar disorder, current episode manic without psychotic features, severe |
|  | F31.2 | Bipolar disorder, current episode manic severe with psychotic features |
|  | F31.30 | Bipolar disorder, current episode depressed, mild or moderate severity, unspecified |
|  | F31.31 | Bipolar disorder, current episode depressed, mild |
|  | F31.32 | Bipolar disorder, current episode depressed, moderate |
|  | F31.4 | Bipolar disorder, current episode depressed, severe, without psychotic features |
|  | F31.5 | Bipolar disorder, current episode depressed, severe, with psychotic features |
| Bipolar and Related Disorders (continued) | F31.60 | Bipolar disorder, current episode mixed, unspecified |
|  | F31.61 | Bipolar disorder, current episode mixed, mild |
|  | F31.62 | Bipolar disorder, current episode mixed, moderate |
|  | F31.63 | Bipolar disorder, current episode mixed, severe, without psychotic features |
|  | F31.64 | Bipolar disorder, current episode mixed, severe, with psychotic features |
|  | F31.70 | Bipolar disorder, currently in remission, most recent episode unspecified |
|  | F31.71 | Bipolar disorder, in partial remission, most recent episode hypomanic |
|  | F31.72 | Bipolar disorder, in full remission, most recent episode hypomanic |
|  | F31.73 | Bipolar disorder, in partial remission, most recent episode manic |
|  | F31.74 | Bipolar disorder, in full remission, most recent episode manic |
|  | F31.75 | Bipolar disorder, in partial remission, most recent episode depressed |
|  | F31.76 | Bipolar disorder, in full remission, most recent episode depressed |
|  | F31.77 | Bipolar disorder, in partial remission, most recent episode mixed |
|  | F31.78 | Bipolar disorder, in full remission, most recent episode mixed |
|  | F31.81 | Bipolar II disorder |
|  | F31.89 | Other bipolar disorder |
|  | F31.9 | Bipolar disorder, unspecified |
|  | F34.0 | Cyclothymic disorder |
| Dissociative Disorders | F44.0 | Dissociative amnesia |
|  | F44.1 | Dissociative fugue |
|  | F44.2 | Dissociative stupor |
|  | F44.81 | Dissociative identity disorder |
|  | F48.1 | Depersonalization-derealization syndrome |
| Mental Health Symptom | R44.0 | Auditory hallucinations |
|  | R44.1 | Visual hallucinations |
|  | R44.2 | Other hallucinations |
|  | R44.3 | Hallucinations, unspecified |
|  | R46.0 | Very low level of personal hygiene |
|  | R46.1 | Bizarre personal appearance |
|  | R46.2 | Strange and inexplicable behavior |

^1^ Diagnosis Group from Child and Adolescent Mental Health Disorders Classification System (CAMHD-CS)

**Supplemental Table 2. Scheme for Categorization of Clinical Significance**

| **Code** | **Clinical  Significance** | **Examples of  Laboratory Studies^1^** | **Examples of  Neuroimaging Studies^1^** |
| --- | --- | --- | --- |
| 1 | Test obtained and normal | - Within laboratory reference range | - No findings |
| 2 | Initial test abnormal and repeat normal without intervention | - Potassium 6.5 (hemolyzed), with repeat potassium 4.5 | - Non-specific abnormality on head computed tomography, with subsequent normal brain magnetic resonance imaging study |
| 3 | Mildly abnormal but did not impact management | - Sodium 131 - Hemoglobin 11.0 - Thyroid stimulating hormone mildly elevated with normal free thyroxine | - Anatomic variants such as small arachnoid cysts - Radiographic evidence of sinusitis that is not clinically treated |
| 4 | Abnormal associated with further testing or treatment but no medical admission | - Hemoglobin 10.0, additional labs sent to screen for iron deficiency anemia - Elevated glucose in a patient with known diabetes, given home dose of insulin | - Leptomeningeal enhancement that prompted lumbar puncture, which was normal |
| 5 | Abnormal, and associated with medical admission  (Note: Only applicable for studies performed the emergency department; code not applied for studies performed after admission) | - Urinalysis with >182 white blood cells in the setting of clinical suspicion for pyelonephritis, admitted for intravenous antibiotics - Creatine kinase 5000, admitted for intravenous fluid hydration | - Brain tumor - Obstructing hydrocephalus - Intracranial hemorrhage - Abscess |
| 6 | Significantly abnormal with no intervention | - Ethanol level 70, no intervention | - Non-depressed skull fracture |

^1^ These examples were provided to data abstractors during training and do not represent

actual patients included in the study.

**Supplemental Table 3. Brief Descriptions of Clinically Significant Neuroimaging Findings among Children and Adolescents Presenting to the Emergency Department with Symptoms of Psychosis**

| **Brief Description of Clinically Significant^1^ Neuroimaging Finding** | **N (%)**  **N=80** |
| --- | --- |
| Non-specific / other findings | 16 (20.0) |
| Mass | 9 (11.3) |
| Pituitary abnormality | 6 (7.5) |
| Meningeal enhancement | 5 (6.3) |
| Dysplasia | 4 (5.0) |
| Acute disseminated encephalomyelitis vs encephalitis | 4 (5.0) |
| Paranasal sinus abnormality | 4 (5.0) |
| Vascular abnormality | 4 (5.0) |
| Abscess or empyema | 4 (5.0) |
| Venous sinus thrombosis | 3 (3.8) |
| Arachnoid cyst | 2 (2.5) |
| Postoperative changes | 2 (2.4) |
| Ventriculomegaly or hydrocephalus | 2 (2.5) |
| Atrophy | 1 (1.3) |
| Cerebellitis | 1 (1.3) |
| Cerebral hyperperfusion | 1 (1.3) |
| Cervical emphysema | 1 (1.3) |
| Choroidal fissure cyst | 1 (1.3) |
| Demyelination | 1 (1.3) |
| Diffuse brain swelling | 1 (1.3) |
| Encephalomalacia | 1 (1.3) |
| Non-diagnostic study | 1 (1.3) |
| Prominent cerebrospinal fluid spaces | 1 (1.3) |
| Prominent perivascular spaces | 1 (1.3) |
| Rathke's cleft cyst | 1 (1.3) |
| Subdural hemorrhage | 1 (1.3) |
| Ventricular asymmetry | 1 (1.3) |
| White matter abnormality | 1 (1.3) |

^1^ Clinically significant findings were defined as those that resulted in further testing, management, or medical admission.

**Supplemental Table 4. Detailed Descriptions of Clinically Significant Neuroimaging Findings among Children and Adolescents Presenting to the Emergency Department with Symptoms of Psychosis**

| **Brief Description** | **Radiology Report Impression** | **Significance** | **Relation to Psychosis** |
| --- | --- | --- | --- |
| Abscess | Prominent lactate peak with depressed NAA peak and no significant increase choline peak. The spectral pattern is more suggestive of an inflammatory process such as an abscess rather than a neoplastic etiology. Correlate clinically. | Associated with further testing or treatment | Likely related |
| Abscess | Rim-enhancing lesion of the left posterior medial temporal lobe with central restricted diffusion and surrounding edema. Findings are most suggestive of an abscess. Other etiologies including neoplasm are considered less likely. Normal MRA and MRV of the head. | Associated with medical admission | Likely related |
| Acute disseminated encephalomyelitis | Widespread, poorly marginated cerebral white matter hypoattenuation and expansion involving the corpus callosum and right greater than left centrum semiovale. Infectious or inflammatory etiology is favored, to include acute disseminated encephalomyelitis (ADEM). Infiltrative neoplasm is also possible, although less consistent with the clinical presentation. | Associated with medical admission | Likely related |
| Acute disseminated encephalomyelitis | 1. Extensive, confluent white matter signal abnormality and mild expansion involving the corpus callosum and bilateral posterior cerebral white matter, with extension into the brainstem and bilateral cerebellar peduncles. Minimal associated diffusion restriction and contrast enhancement. 2. Extensive signal abnormality and mild expansion of nearly the entire length of the spinal cord, involving the central cord and posterior columns. 3. An inflammatory process such as ADEM is favored, although the lesions are more extensive and confluent than typical. Acute infectious encephalomyelitis is also possible. | Associated with medical admission | Likely related |
| Acute disseminated encephalomyelitis vs encephalitis | 1. Extensive T2 signal abnormality and mild expansion involving the bilateral posterior cerebral white matter, brainstem, and cerebellar peduncles, minimally decreased in extent since the prior exam. Few small internal foci of diffusion restriction and contrast enhancement are unchanged. No new lesions are identified. 2. Moderate lactate peak on MR spectroscopy of the parietal white matter. Normal MR spectroscopy of the basal ganglia. 3. Differential includes ADEM and infectious encephalitis. Presence of lactate in the white matter is nonspecific, but also raises the possibility of a metabolic leukoencephalopathy such as LBSL. | Associated with medical admission | Likely related |
| Arachnoid cyst | 1. Large left middle cranial fossa arachnoid cyst with extension into the anterior cranial fossa, not significantly changed compared to prior CT. Stable chronic appearing intracranial mass effect as detailed. 2. Brain parenchyma is normal in signal intensity. No abnormal contrast enhancement. | Associated with further testing or treatment | Unclear |
| Arachnoid cyst | Findings, as described, are consistent with left middle cranial fossa arachnoid cyst. | Associated with medical admission | Unclear |
| Atrophy | 1. Mild symmetric prominence of ventricles and sulci, likely reflecting mild diffuse brain parenchymal atrophy and/or pseudoatrophy. 2. Clear paranasal sinuses. | Associated with further testing or treatment | Unclear |
| Cerebellitis | 1. Findings of diffuse cerebellitis characterized by cortical edema, leptomeningeal enhancement and increased perfusion. Basal cisterns are patent. Increased perfusion of the cerebrum, seizure versus encephalitis. 2. Stable appearance of bilateral lateral, third and fourth ventricle compared to prior CT. | Associated with further testing or treatment | Likely related |
| Cerebral hyperperfusion | 1. Diffuse cerebral hyperperfusion. Additionally, diffuse T2 hyperintensity signal of the subarachnoid spaces. 2. In the proper clinical setting the findings may represent an infiltrative process such as meningoencephalitis. 3. Enlargement of the pituitary gland, at the upper limits of normal for a pubertal gland. No discrete lesion is however identified. | Associated with medical admission | Likely related |
| Cervical emphysema | Deep cervical emphysema, similar to demonstrated on chest radiograph from earlier today. Otherwise negative head CT without contrast. | Associated with medical admission | Likely unrelated |
| Choroidal fissure cyst | 1. 8 mm rounded hypoattenuating focus in the right mesial temporal region most likely representing a small choroidal fissure cyst, but incompletely characterized by noncontrast head CT. Nonemergent follow up with MRI may be considered, as clinically indicated. 2. Otherwise unremarkable noncontrast CT examination of the head, with no acute intracranial abnormality identified. | Associated with further testing or treatment | Likely unrelated |
| Demyelination | No change in the signal abnormalities involving the bilateral periventricular white matter. No new lesions are identified. Findings could reflect dysmyelination or possibly demyelinating process. No evidence of other intracranial abnormality. | Associated with medical admission | Unclear |
| Diffuse brain swelling | Diffuse brain swelling. | Associated with medical admission | Likely related |
| Dysplasia | 1. No acute intracranial abnormality. 2. Focal thinning of the posterior body of the corpus callosum, which may represent mild callosal dysplasia. | Associated with further testing or treatment | Likely unrelated |
| Dysplasia | Focal anterior right temporal lesion, suspect cortical dysplasia | Associated with medical admission | Likely related |
| Dysplasia | Suspected focal cortical dysplasia involving the left temporal pole. No mesial temporal sclerosis. The brain is otherwise unremarkable. | Associated with medical admission | Unclear |
| Dysplasia | 1. Transmantle heterotopia involving the right temporal and occipital lobes, with associated polymicrogyria along the right superior temporal sulcus. 2. Restricted diffusion in the splenium of the corpus callosum. Finding is nonspecific and may be secondary to seizure activity or infectious/postinfectious encephalitis. | Associated with medical admission | Unclear |
| Empyema | 1. A 2 to 3 mm thick subdural fluid collection seen over the left frontal and temporal lobes, extending medially along the falx cerebri. This is concerning for a subdural empyema. There is mild mass effect on underlying parenchyma with mild medial deviation of the uncus at the level of suprasellar cistern. 2. No parenchymal abscess identified. No ventriculomegaly. 3. Left-sided paranasal sinus disease with air-fluid levels, could be related to sinusitis. The middle ear and mastoids are clear. | Associated with medical admission | Likely related |
| Empyema | 1. Extra-axial collection and enhancement overlying the left cerebral hemisphere, most pronounced over the left frontal lobe, measuring approximately 3 mm in maximal thickness, highly suspicious for subdural empyema. There is associated mild mass effect upon the underlying brain parenchyma, and extension of enhancement and signal abnormality into the underlying sulci. 2. Limited examination due to susceptibility artifact from orthodontic braces, precluding evaluation of the paranasal sinuses, limited evaluation of the inferior frontal regions, and rendering the diffusion weighted images nondiagnostic. 3. Normal ventricular size and configuration. | Associated with medical admission | Likely related |
| Encephalitis | Allowing for pronounced motion artifacts, there may be a subtly increased signal hyperintensity of the mesial temporal lobes bilaterally. Autoimmune limbic encephalitis can be considered as a potential differential diagnosis, but changes may also be non-specific. Otherwise, normal MR imaging appearance of the brain. | Associated with medical admission | Likely unrelated |
| Encephalomalacia | 1. No acute intracranial abnormality. 2. Focal encephalomalacia in the right occipital lobe, which may be secondary to prior infarct, trauma, or infection. | Associated with further testing or treatment | Unclear |
| Hydrocephalus | Moderate to severe hydrocephalus slightly decreased since prior. | Associated with medical admission | Likely related |
| Mass | MR brain: 1. Complex cystic and enhancing solid cerebellar mass lesion with intracystic hemorrhage and surrounding edema. Imaging features are most suggestive of pilocytic astrocytoma, possibly pilomyxoid variant given hemorrhage. 2. Persistent but slightly decreased moderate obstructive hydrocephalus following right frontal ventriculostomy placement. | Associated with further testing or treatment | Likely related |
| Mass | 1. No hydrocephalus or acute ischemia or recent intracranial hemorrhage by limited rapid MR. 2. Partially visualized T2 hypointense expansile right nasoethmoidal mass. This requires additional imaging with MR and/or CT for characterization. | Associated with further testing or treatment | Likely unrelated |
| Mass | Large heterogeneous mass centred in the suprasellar-left hypothalamic region with extension as described with moderate obstructive hydrocephalus (right more than left). Features are suggestive of higher grade glioma. The spine has not been optimally evaluated due to motion and will have to be repeated. | Associated with medical admission | Likely related |
| Mass | Mixed solid and cystic mass in the posterior fossa, centered in the lower aspect of the cerebellum/posterior foramen magnum region with mass effect on the fourth ventricle and secondary hydrocephalus. No hemorrhage or calcification identified. The solid aspect of the lesion is isodense to cortical tissue. Diagnostic considerations should include cystic medulloblastoma, a pilocytic astrocytoma, less likely an ependymoma. | Associated with medical admission | Likely related |
| Mass | There is a small mass lesion in the region of the tectum with high T2 signal without significant enhancement suggesting a possible tectal glioma. There is associated obstructive hydrocephalus at the level of the aqueduct with dilatation of the lateral and third ventricles and transependymal flow of CSF. No midline shift. | Associated with medical admission | Likely related |
| Mass | 1.5 cm increased density lesion which appears to be in the left lateral ventricle. Entities such as ependymoma or subependymoma is considered. Overproduction of CSF is not evident to suggest choroid plexus papilloma. Intraventricular meningioma is not excluded. Focal hemorrhage may be less likely. MRI may be helpful for further evaluation. | Associated with medical admission | Likely related |
| Mass | Additional high resolution and neuronavigational sequences obtained to complement the recently done MRI study. The pituitary gland is identified separately and the relationship with the optic chiasm is better seen, as described (the optic chiasm is displaced anteriorly and to the right. The left side of the optic chiasm, intracranial optic nerve and tract can be identified but are intimately related to the tumor. Evidence of bilateral papilledema noted. The lateral ventricles are dilated, right more than left with signs of transependymal edema). | Associated with medical admission | Likely related |
| Mass | Features felt to represent an intraventricular mass lesion arising within the temporal horn left lateral ventricle or along the ventricular wall. The presence of fine calcification on the head CT would suggest a lesion such as a choroid plexus papilloma or meningioma; however, the lesion did not have margination typical for a choroid plexus papilloma. Also choroid plexus papillomas and meningiomas generally markedly enhance-this lesion did not. Therefore, the differential could be expanded to include central neurocytoma, ependymoma, subependymoma or other lower grade glial tumor. | Associated with medical admission | Likely related |
| Mass | 1. Single nonspecific 9 mm cystic focus in or adjacent to the superficial right parietal lobe without mass effect or parenchymal edema. This could be further characterized with MRI on a nonemergent basis if clinically indicated. 2. Otherwise normal noncontrast head CT. | Associated with medical admission | Unclear |
| Meningeal enhancement | New mild cerebellar leptomeningeal enhancement, and evidence of worsened cerebellar swelling with worsening of downward tonsillar extension and mass effect on the cervicomedullary junction in the setting of a pre-existing complex Chiari malformation. Probable slight worsening in subtle enhancement of multiple cranial nerves. Findings are suggestive of a infectious or inflammatory cerebellitis/encephalitis, recommend clinical correlation. | Associated with medical admission | Likely related |
| Meningeal enhancement | 1. No evidence of intracranial abscess, empyema, cerebritis or infarction. 2. However noted increased cerebral perfusion and areas of increased T2 prolongation cerebral gyri. Findings, in the setting of reported clinical concern for encephalitis, might be indicative of leptomeningeal process such as meningitis. Clinical correlation is advised. | Associated with medical admission | Likely related |
| Meningeal enhancement | Possible subtle leptomeningeal enhancement in the parietal convexities may represent meningitis in this clinical setting. Clinical correlation please. No other acute intracranial abnormality. | Associated with medical admission | Likely related |
| Meningeal enhancement | Diffuse pachymeningeal enhancement over both cerebral hemispheres, new from the prior exam. Appearance is nonspecific but can be seen with intracranial hypotension. Air-fluid levels in the paranasal sinuses suggesting sinusitis. No evidence of toxic leukoencephalopathy or posterior reversible encephalopathy syndrome. | Associated with medical admission | Likely related |
| Meningeal enhancement | Thick leptomeningeal enhancement seen throughout the basal cisterns and the surface of the cervical spinal cord. Small extra-axial CSF signal fluid collections are seen in bilateral cerebellopontine and cerebellomedullary cisterns. These findings could be related to fungal or atypical (including tuberculous) meningitis. Other less likely etiologies for this imaging appearance could be neoplastic process or neurosarcoidosis. 2. Moderate dilatation of the ventricular system with mild periventricular interstitial edema, suggestive of communicating hydrocephalus. There is diffuse effacement of the cerebral sulci and partial effacement of the suprasellar cistern. 3. No parenchymal ischemia or abscess identified. | Associated with medical admission | Likely related |
| Non-specific / other findings | Ill defined very small focus of intermediate attenuation in the region of the hypothalamus, anterior inferior to the expected location of the mamillary bodies, without mass effect on adjacent structures. Potential differential diagnosis includes small hamartoma of the tuber cinereum or other mass in this region, less likely a hypothalamic fusion anomaly. Recommend MRI of the brain with contrast for further evaluation. Patient was tearful and intermittently not cooperative in the CT scanner, therefore recommend that this further imaging be performed with sedation/anesthesia. 2. Opacified left maxillary sinus with moderate mucosal thickening in the right maxillary sinus and right sphenoid sinus. | Associated with further testing or treatment | Likely unrelated |
| Non-specific / other findings | A punctate focus of restricted diffusion in the right cerebellum for which the differential includes small cerebellar infarct versus sequalea of infection/inflammation or demyelinating disease. | Associated with further testing or treatment | Likely unrelated |
| Non-specific / other findings | 12 mm nonenhancing focus of T2 hyperintensity adjacent to the left frontal horn without restricted diffusion or mass effect, nonspecific. 6 month follow-up brain MRI would be helpful to confirm stability. Otherwise normal MRI of the brain without and with contrast. | Associated with further testing or treatment | Likely unrelated |
| Non-specific / other findings | 1. Focal hyperdensity with minimal calcification is noted in the region of roof of third ventricle. This could represent a small colloid cyst or a focal vascular calcification. Other abnormalities like a neoplastic process cannot be totally excluded. Evaluation by an MR scan can be done. | Associated with further testing or treatment | Unclear |
| Non-specific / other findings | 1. Interval small foci of calcification of the globus pallidus, bilaterally. 2. Etiologies include calcium phosphate metabolism disorder, carbon monoxide poisoning, metabolic disorders, infectious processes among other other processes. | Associated with further testing or treatment | Unclear |
| Non-specific / other findings | Increased T2 FLAIR and enhancement signal of the temporal parietal and occipital subarachnoid spaces. Findings likely due to sedation rather than indicative of leptomeningeal disease. However clinical correlation and or lumbar puncture is advised for further evaluation. | Associated with further testing or treatment | Unclear |
| Non-specific / other findings | 1. No evidence of acute infarct. 2. Probable chronic injury in the right occipital lobe. | Associated with further testing or treatment | Unclear |
| Non-specific / other findings | Small ill-defined hyperdensity with associated calcifications in the superficial portion of the right temporal lobe. Differential diagnosis includes a cavernous malformation or other vascular malformation, low-grade neoplasm, or dysplasia. Lesional hyperattenuation at least in part represents calcification; however, age indeterminate blood products cannot be entirely excluded. Follow-up MRI of the brain without with contrast and MR angiogram is recommended for further evaluation. | Associated with medical admission | Likely related |
| Non-specific / other findings | There is a right frontal subcortical hyperdensity with CT density around 60 HU, measuring 15 mm nonspecific but may be related to intraparenchymal/subarachnoid bleeding or hyperdense mass. | Associated with medical admission | Likely related |
| Non-specific / other findings | There is no intracranial mass lesion, diffusion restriction or abnormal parenchymal enhancement noted. Some tiny nonspecific foci of FLAIR/T2 hyperintensity are seen in the frontal white matter bilaterally. If a CNS infection or neuroinflammatory process is suspected clinically, CSF correlation is recommended. There are tiny outpouchings seen arising from the proximal P1 segments of the posterior cerebral arteries bilaterally (left greater than right) favored to represent bilateral infundibuli rather than tiny aneurysms. | Associated with medical admission | Likely related |
| Non-specific / other findings | 1. Focus of left parieto-occipital cortical thickening and signal abnormality, as discussed. Differential considerations include focal cortical dysplasia, low-grade glial tumor versus seizure related change or cerebritis. Recommend correlation with CSF analysis and close surveillance imaging (2-3 months). 2. More questionable cortical thickening and signal abnormality of the adjacent left temporal and parieto-occipital cortex is favored to be artifactual. | Associated with medical admission | Likely related |
| Non-specific / other findings | Findings consistent with a remote injury of the white matter in the right cerebral hemisphere as described. There is no evidence for an acute intracranial event. | Associated with medical admission | Likely related |
| Non-specific / other findings | Subtle T2 and FLAIR increased signal intensity of the periventricular white matter along with widened sulci, ventricles and basal cisterns of the brain which are probably treatment related. Otherwise the gray-white matter differentiation is preserved. No evidence of on-going brain lesion. | Associated with medical admission | Likely related |
| Non-specific / other findings | No significant change in the small nonspecific foci of increased FLAIR signal in the supratentorial white matter. No evidence of contrast enhancement to suggest an active inflammatory process | Associated with medical admission | Likely related |
| Non-specific / other findings | A few small foci of supratenteorial white matter FLAIR hyperintensity which are non-specific. Otherwise unremarkable MRI or the brain. If a neuroinflammatory or encephalitic disorder is suspected the patient could return for a contrast enhanced MRI as warranted after review by neurology | Associated with medical admission | Likely unrelated |
| Non-specific / other findings | Nonspecific focus of ill-defined hypodensity/edema in the posterior medial left temporal lobe. This could be further characterized utilizing MRI brain with and without contrast. | Associated with medical admission | Unclear |
| Non-diagnostic study | Motion degraded, limited, nondiagnostic study. No evidence of acute infarct, large hemorrhage or mass effect. Axial FLAIR images are significantly motion degraded, and nondiagnostic. Follow-up brain MRI with sedation and adequate FLAIR sequence is recommended if there is continued concern for infectious or inflammatory process. Post-contrast images are also significantly motion degraded and nondiagnostic. No gross abnormal enhancement is seen. | Associated with further testing or treatment | Likely unrelated |
| Paranasal sinus abnormality | 1. No hydrocephalus. Limited evaluation of the parenchyma. 2. Extensive paranasal sinus and bilateral mastoid opacification | Associated with further testing or treatment | Likely related |
| Paranasal sinus abnormality | No evidence of intracranial hemorrhage, mass effect or large acute territorial infarct. If clinical neurological symptoms persist, MRI is a more sensitive examination. Mild partially visualized paranasal sinus mucosal inflammatory thickening. | Associated with further testing or treatment | Likely unrelated |
| Paranasal sinus abnormality | 1. Left-sided sinonasal opacification in ostiomeatal and sphenoethmoidal recess patterns without definite underlying bony lesion. 2. Normal intracranial contents | Associated with further testing or treatment | Unclear |
| Paranasal sinus abnormality | No acute intracranial abnormality. Opacification of the paranasal sinuses with fluid level in the maxillary sinuses and complete opacification of the left sphenoid sinus. Correlate for acute sinusitis. | Associated with further testing or treatment | Unclear |
| Pituitary abnormality | There is enlargement of the anterior pituitary gland beyond what would be expected for physiologic hyperplasia. Finding is nonspecific and incompletely assessed without dedicated pituitary sequences, though considerations could include nonphysiologic hyperplasia, versus underlying primary pituitary lesion such as adenoma or pituitary cyst also not absolutely excluded. Consider dedicated pituitary postcontrast sequences for further examination of the sella. | Associated with further testing or treatment | Likely unrelated |
| Pituitary abnormality | 1. Convex superior margin to enlarged pituitary gland which demonstrates uniform enhancement. Correlation with endocrine symptoms is recommended as for the need for dedicated sellar evaluation for better further evaluation and to exclude adenoma. 2. CSF isointense spaces of the bilateral ventral basal ganglia largest if which is seen on the right. These are most consistent with prominent perivascular spaces. 3. Otherwise unremarkable contrasted MRI of the brain. | Associated with further testing or treatment | Likely unrelated |
| Pituitary abnormality | Dedicated postcontrast pituitary imaging redemonstrates significant enlargement of the anterior pituitary gland beyond dimensions would be expected for physiologic hyperplasia. There is no evidence of a focal pituitary lesion or focal region of hypoenhancement. The imaging appearance is nonspecific but could correspond with nonphysiologic hyperplasia, which can be associated with neuroendocrine disorder, hypothyroid disorder, or Addison disease, with alternate etiologies not absolutely excluded | Associated with further testing or treatment | Likely unrelated |
| Pituitary abnormality | The pituitary measures at the upper limits for size and appears slightly hyperdense, with thickening of the infundibulum. There is mild expansion and thinning of the sella turcica. The ventricles and extra-axial spaces are normal in size and configuration. Brain parenchymal attenuation is normal. There is no intracranial hemorrhage. The osseous structures appear normal. The imaged sinuses are clear. | Associated with further testing or treatment | Unclear |
| Pituitary abnormality | Normal noncontrast head CT. 2. A small pituitary lesion cannot be excluded on a noncontrast head CT, and nonemergent contrast MRI should be considered if clinically concerned. | Associated with further testing or treatment | Unclear |
| Pituitary abnormality | 2 mm hypoenhancing lesion within the pituitary may reflect a pituitary microadenoma versus pars intermedia cyst. Consider pituitary protocol MRI for further characterization. Otherwise no acute intracranial abnormality. | Associated with medical admission | Likely unrelated |
| Postoperative changes | 1. Status post placement of a right frontal approach external ventricular drain catheter with expected postoperative changes. 2. Persistent moderate global hydrocephalus, not significant changed | Associated with further testing or treatment | Likely related |
| Postoperative changes | Status post VP shunt insertion with postoperative changes including pneumocephalus and mild intraventricular hemorrhage. There has been interval decrease in ventricular size compared to the prior examination. | Associated with medical admission | Likely related |
| Prominent cerebrospinal fluid spaces | Compared to prior, CSF spaces are mildly more prominent. | Associated with medical admission | Likely related |
| Prominent perivascular spaces | Mild symmetric hypodensity in the bilateral superior frontal white matter. Findings likely represent prominent perivascular spaces, a normal variant, although white matter edema or gliosis is not excluded. Consider noncontrast brain MRI for further evaluation | Associated with further testing or treatment | Likely unrelated |
| Rathke's cleft cyst | Findings are suspicious for a Rathke's cleft cyst. However, a pituitary gland adenoma is not excluded, and pituitary gland function blood test is helpful. | Associated with further testing or treatment | Likely unrelated |
| Subdural hemorrhage | Thin layering posterior falx and left tentorial subdural hemorrhage not seen on the prior. Otherwise unchanged compared to prior. | Associated with medical admission | Likely related |
| Vascular abnormality | 1. Right temporal cavernous malformation, as above, corresponding to hyperdensity on recent CT. 2. Relative hypoperfusion in the right anterior temporal lobe, which is nonspecific but could relate to seizure activity. 3. Normal MRA of the major intracranial arteries. | Associated with further testing or treatment | Likely related |
| Vascular abnormality | An amorphous hyperdensity in the right frontal parasagittal centrum semiovale likely represents a small region of hemorrhage, less likely mineralization or hypercellular lesion. If hemorrhage, vascular malformation or hemorrhagic neoplasm would be more likely than trauma. MR brain is advised for further evaluation. | Associated with further testing or treatment | Likely unrelated |
| Vascular abnormality | 1. Apparent small fenestration in the distal basilar artery, not well evaluated on this noncontrast MRI. Further characterization of this finding with noncontrast MRA of the head may be considered, if clinically indicated. 2. Otherwise unremarkable noncontrast MRI examination of the brain, with no acute intracranial abnormality identified. | Associated with further testing or treatment | Likely unrelated |
| Vascular abnormality | 1.4 cm mixed intensity lesion in the right frontal lobe, most cmpatible with a cavernoma. There is a prominent hemosiderin ring and various stages of associated hemorrhage. There is no surrounding edema, midline shift, herniation, or hydrocephalus. There are two additional punctate foci in the left parietal lobe and right cerebellar hemisphere which are also suspicious for cavernomas (cavernomatosis), which may be a hereditary/familial. | Associated with medical admission | Likely related |
| Venous sinus thrombosis | Intraparenchymal multifocal hemorrhage along the right parietotemporal occipital lobes with prominent surrounding low attenuation and suspected hyperdensity along the right sigmoid sinus suggestive of possible venous sinus thrombosis and associated venous infarction with hemorrhage conversion. CTA/CTV head is recommended for further evaluation. | Associated with medical admission | Likely related |
| Venous sinus thrombosis | No arterial abnormality is demonstracted. Findings highly concerning for acute dural venous sinus thrombosis involving the right transverse and sigmoid sinuses extending into the right jugular vein. | Associated with medical admission | Likely related |
| Venous sinus thrombosis | Occlusive dural venous sinus thrombosis involving the right transverse and sigmoid sinus extending into the upper right internal jugular vein. | Associated with medical admission | Likely related |
| Ventricular asymmetry | No evidence of intracranial hemorrhage, mass effect or large acute infarct. The right lateral ventricle is larger than the left, without evidence of transependymal CSF. Consider follow-up non urgent brain MRI for complete evaluation as clinically indicated. | Associated with medical admission | Unclear |
| Ventriculomegaly | Moderate global ventriculomegaly, which is disproportionate to the cortical sulci, raises concern for increased intracranial pressure. Correlate clinically. Mild increase in T2/ FLAIR signal in the splenium of the corpus callosum. | Associated with medical admission | Likely related |
| White matter abnormality | Lower than expected density of the white matter throughout the brain, which may be a sign of underlying leukoencephalopathy versus artifact. MRI is recommended for further evaluation | Associated with further testing or treatment | Unclear |

**Supplemental Table 5. Interrater Reliability of Data Abstraction of Clinical Characteristics**

| **Characteristic** | **Kappa**  **(95% CI)** |
| --- | --- |
| Age | 0.98 (0.95, 1.00) |
| Male sex | 0.96 (0.90, 1.00) |
| Race and ethnicity | 0.93 (0.86, 1.00) |
| Symptoms of psychosis |  |
| Hallucinations | 0.84 (0.68, 0.99) |
| Delusions | 0.58 (0.36, 0.79) |
| Disorganized speech | 0.63 (0.44, 0.81) |
| Disorganized behavior | 0.65 (0.49, 0.81) |
| Catatonia | 0.94 (0.82, 1.00) |
| Clinical history |  |
| Head trauma | 0.87 (0.69, 1.00) |
| Headache | 0.63 (0.45, 0.81) |
| Seizure-like activity | 0.63 (0.30, 0.96) |
| Amnesia | 0.36 (-0.20, 0.93) |
| Vomiting | 0.91 (0.80, 1.00) |
| Tired/lethargic | 0.66 (0.38, 0.94) |
| Fever (by history) | 0.84 (0.63, 1.00) |
| Alcohol/Substance use | 0.58 (0.25, 0.91) |
| First episode | 0.52 (0.30, 0.75) |
| Weakness | 0.31 (-0.16, 0.79) |
| Sensory | 0.79 (0.39, 1.00) |
| Vision change | 0.57 (0.26, 0.87) |
| Gait change | 0.50 (0.13, 0.88) |
| Past history |  |
| Neurologic condition | 0.62 (0.39, 0.86) |
| Psychiatric condition | 0.62 (0.47, 0.78) |
| Physical exam |  |
| Fever during visit | 1.00 (1.00, 1.00) |
| Tachycardia | 0.86 (0.75, 0.98) |
| Altered consciousness | 0.50 (0.26, 0.74) |
| Meningismus | 1.00 (1.00, 1.00) |
| Focal neurologic deficit | 0.39 (-0.15, 0.93) |
| Disposition | 0.91 (0.84, 0.98) |

**Supplemental Table 6. Factors Associated with Clinically Significant Neuroimaging among Children and Adolescents Presenting to the Emergency Department with Symptoms of Psychosis, Univariate Models**

|  | **OR (95% CI)** | |
| --- | --- | --- |
| **Characteristic** | **Model Using Imputation** | **Model with Missing Values Treated as Absent^1^** |
| Age |  |  |
| 5-10 | 2.62 (1.40, 4.91) | 2.62 (1.40, 4.91) |
| 11-14 | 1.35 (0.71, 2.57) | 1.35 (0.71, 2.57) |
| 15-17 | Ref. | Ref. |
| Male sex | 1.37 (0.80, 2.33) | 1.37 (0.80, 2.33) |
| Hallucinations | 0.59 (0.32, 1.07) | 0.57 (0.34, 0.97) |
| Delusions | 0.93 (0.49, 1.80) | 0.83 (0.45, 1.53) |
| Disorganized speech | 1.22 (0.68, 2.19) | 1.16 (0.66, 2.05) |
| Disorganized behavior | 1.20 (0.69, 2.06) | 1.25 (0.74, 2.11) |
| Catatonia | 1.63 (0.71, 3.72) | 1.61 (0.71, 3.67) |
| Head Trauma | 0.78 (0.18, 3.31) | 0.65 (0.16, 2.75) |
| Vomiting | 1.69 (0.74, 3.86) | 1.68 (0.74, 3.83) |
| Lethargy | 2.65 (1.34, 5.23) | 2.08 (1.10, 3.96) |
| Fever (by history or in the ED) | 2.39 (1.04, 5.52) | 2.41 (1.05, 5.54) |
| Alcohol or substance use | 0.69 (0.28, 1.66) | 0.59 (0.25, 1.40) |
| First-episode psychosis | 2.17 (1.04, 4.50) | 2.42 (1.33, 4.39) |
| History of psychiatric condition | 0.50 (0.29, 0.86) | 0.53 (0.31, 0.92) |
| History of neurologic condition | 0.75 (0.29, 1.92) | 0.78 (0.30, 1.98) |
| Tachycardia | 1.34 (0.75, 2.40) | 1.29 (0.72, 2.30) |
| Altered consciousness | 1.48 (0.80, 2.76) | 1.49 (0.80, 2.78) |
| Neurological signs or symptoms^2^ | 2.37 (1.20, 4.66) | 2.37 (1.20, 4.66) |

^1^ Missing values (with no documentation of the clinical finding within clinician notes) were treated as absent in this model.

^2^ Neurological signs or symptoms included: headache, seizure, amnesia, weakness, sensory, vision change, gait change, meningismus, or focal neurologic deficit. A composite variable was used in models due to few positive cases (<5% of the study sample) for some individual neurologic signs and symptoms.
